# Supplementary figures and images for: Lipid-laden endothelial cells exhibit a transcriptomic signature linked to blood–brain barrier dysfunction, metabolic reprogramming, and increased inflammation in the aging brain
Source: GeroScience. 2026 Jan 16;48(2):1735–50. doi: 10.1007/s11357-025-01986-y (PMC12972456; doi:10.1007/s11357-025-01986-y)

## Suppl Fig 1.

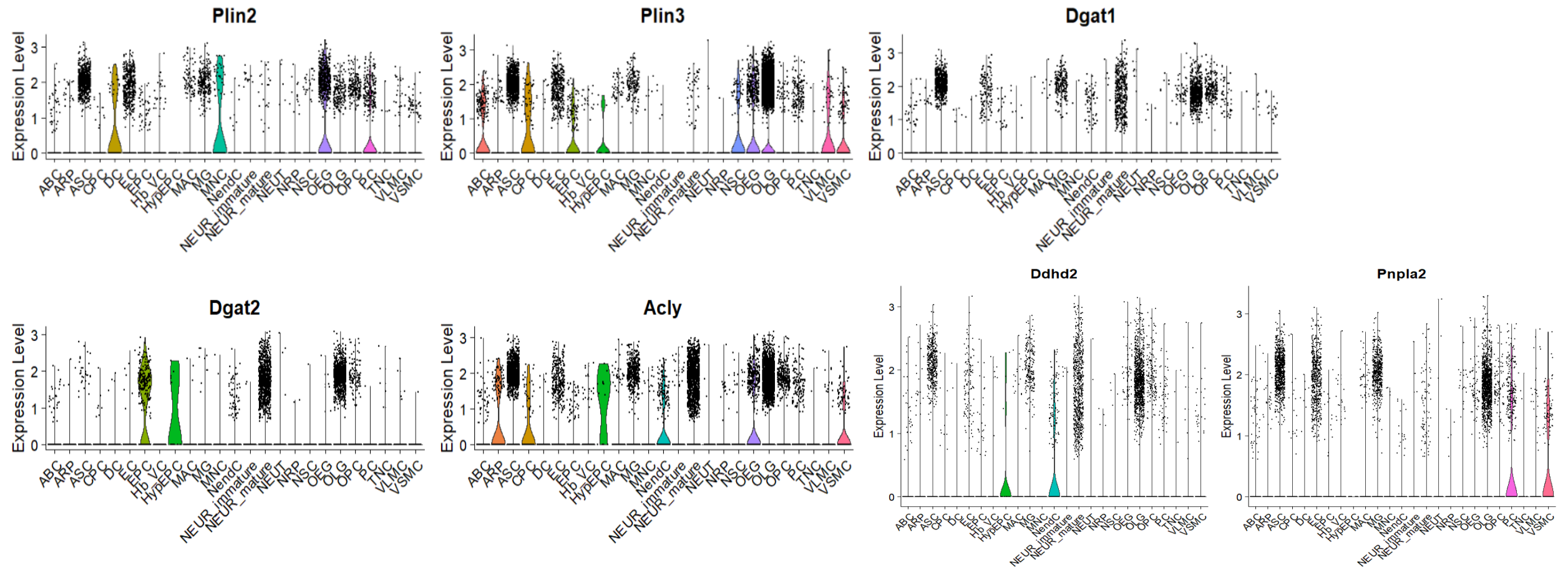

Suppl. Fig. 2

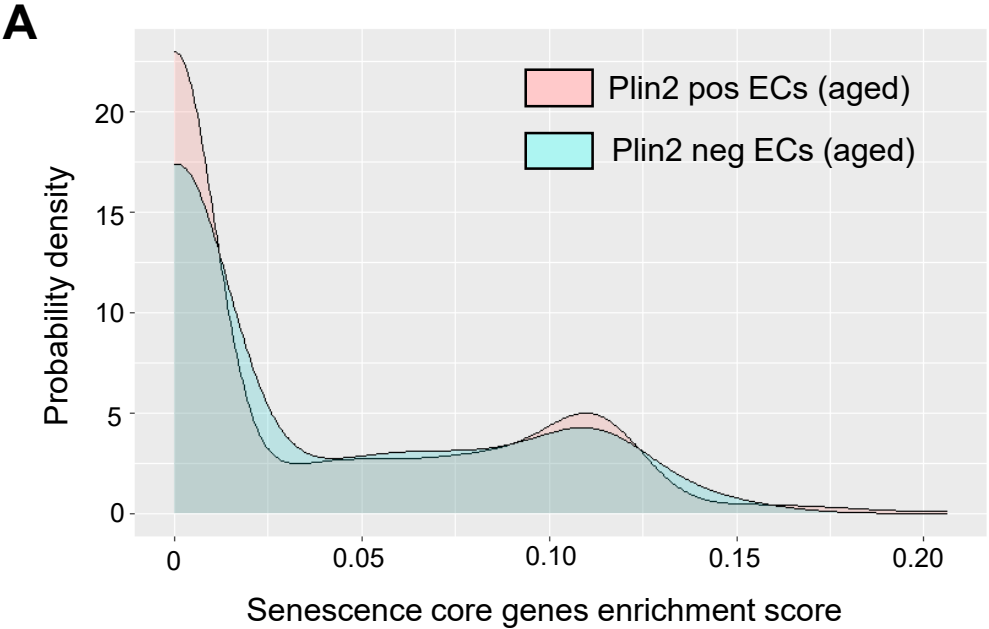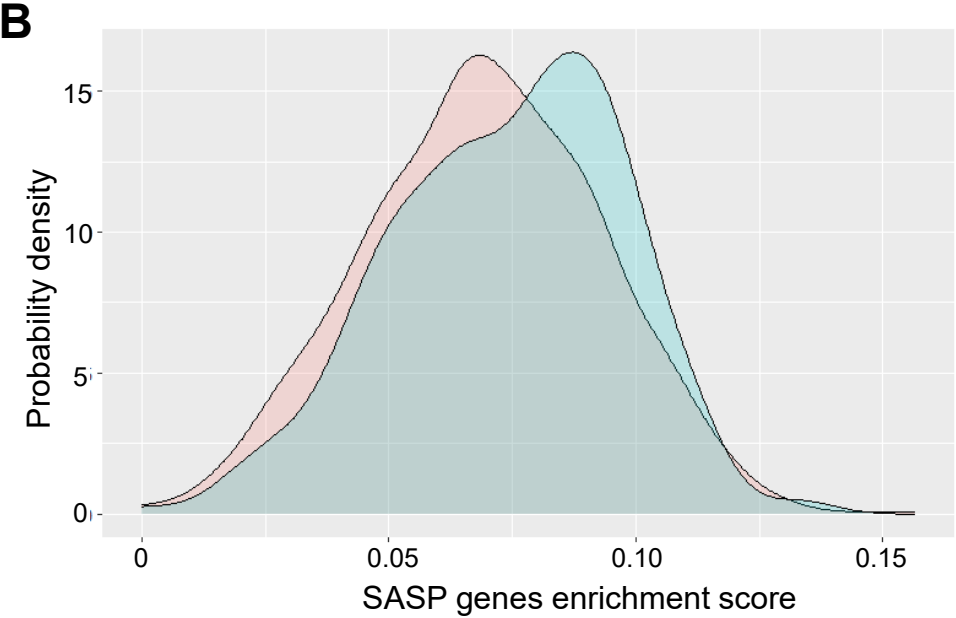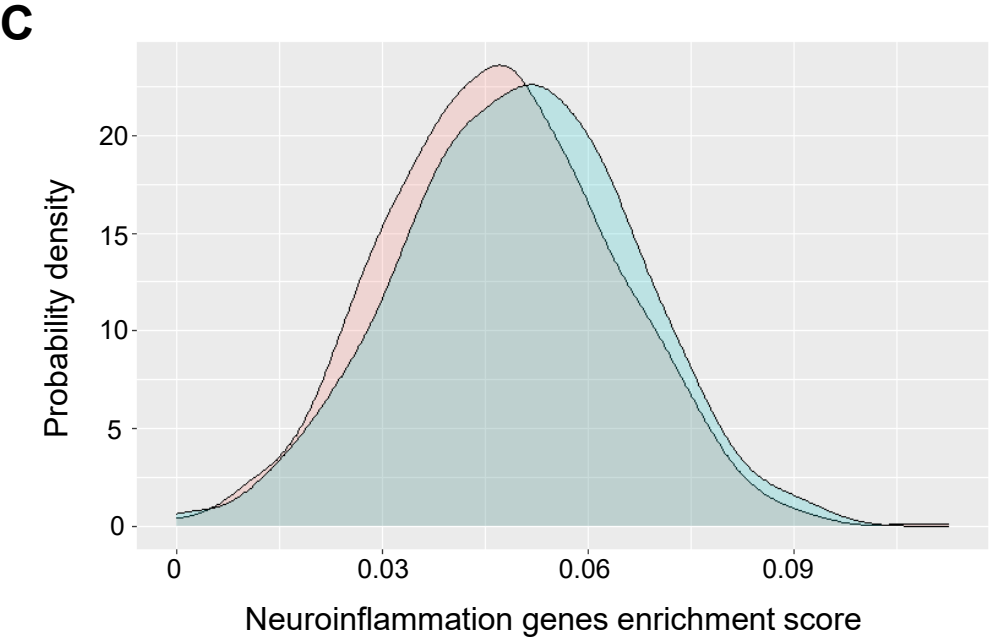

Suppl. Fig. 3

Astrocytes in the aging brain

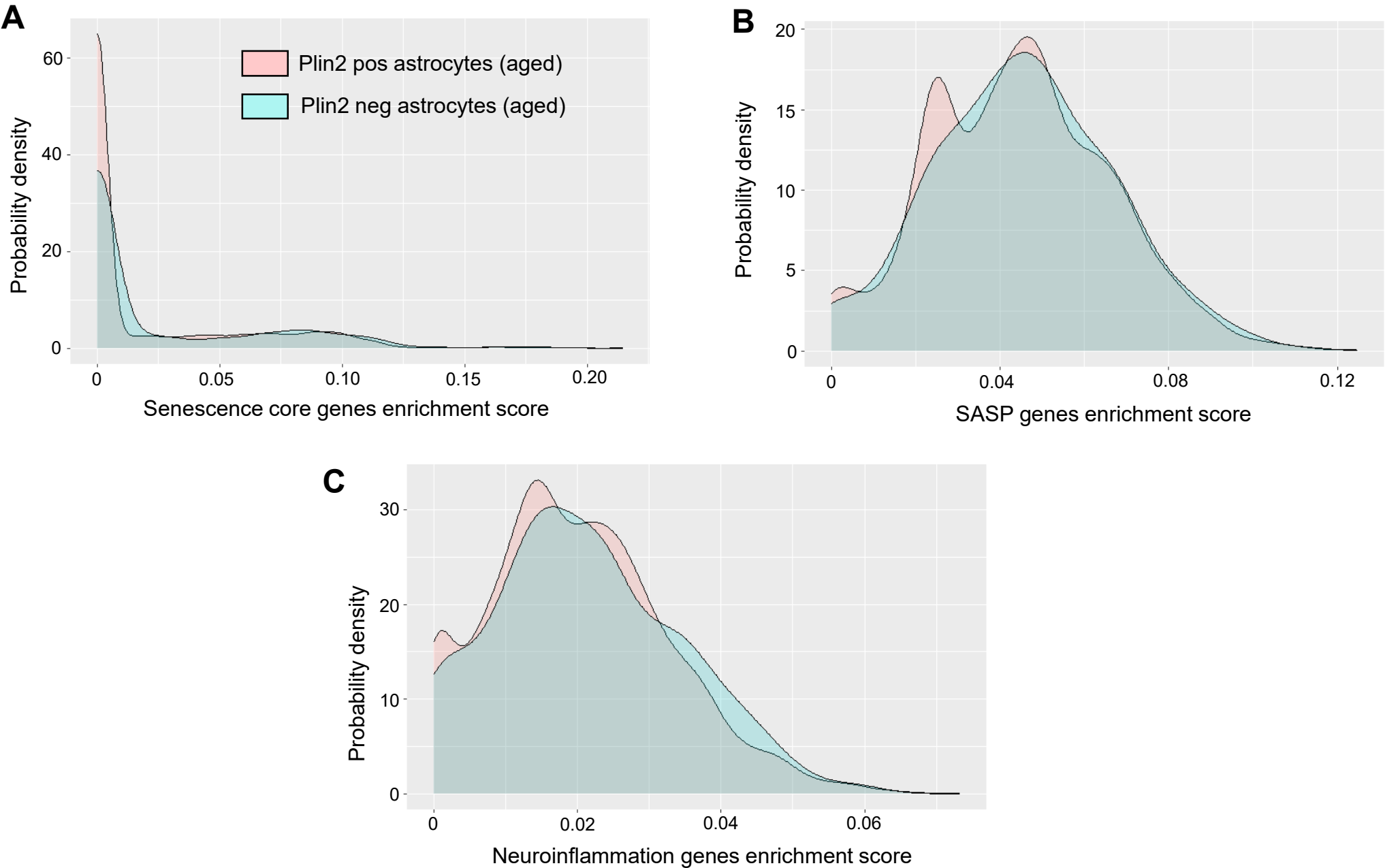

Supplement: Supplementary file 1 — (PDF 435 KB) [file 11357_2025_1986_MOESM1_ESM.pdf]
